# Case report: renal cell carcinoma metastasis to the tongue

**DOI:** 10.1093/jscr/rjac565

**Published:** 2022-12-20

**Authors:** Michael A Walsh, Alanna J Quinn, Bangalore Mahesh

**Affiliations:** Royal College of Surgeons Ireland, Dublin, Ireland; Department of Otolaryngology, University Hospital Waterford, Waterford, Ireland; Royal College of Surgeons Ireland, Dublin, Ireland; Department of Otolaryngology, University Hospital Waterford, Waterford, Ireland; Department of Otolaryngology, University Hospital Waterford, Waterford, Ireland

**Keywords:** Tongue, Renal cell carcinoma, Metastasis

## Abstract

Tongue metastasis of renal cell carcinoma (RCC) are rare, with 90% of malignant lesions in the oral cavity consisting of squamous cell carcinoma (SCC). There are a number of subtypes of SCC which need to be considered as well as rarer pathology and even benign conditions. The case involves a 63-year-old gentleman with an incidental finding of pulmonary metastasis from a RCC of the left kidney, and he concurrently had a RCC deposit on the dorsum of the tongue, which was diagnosed after biopsy. Treatment modalities of surgical excision, radiotherapy and systemic therapy are considered, but with such a poor prognosis with disseminated disease, options are limited. Treatment should include multidisciplinary team input, with a focus on reducing disease morbidity.

## INTRODUCTION

Renal cell carcinoma (RCC) accounts for 2.2% of malignant neoplasms in adults, typically presenting with flank pain, haematuria and flank mass [1]. Metastases of RCC to the head and neck region are rare. The majority of metastasis will seed to the lung (75%), soft tissues (36%), bone (18%) cutaneous and nerve (8%) [2]. While 15% account for deposition in the head and neck, the predominant sites in the region include the paranasal sinus, larynx, jaw and temporal bone [3]. When the oral cavity is the site of metastasis, the primary locations for discovery usually consist of the ostium of the jaw and gingiva [4]. Therefore, considering a tongue lesion as a metastasis is the exception to the rule, with most lesions found here classified as primary oral cavity tumours.

Oral cavity tumours are the most common head and neck cancers, with 90% of these neoplasms consisting of squamous cell carcinoma (SCC), minor salivary gland malignancies and other rare tumours comprising the rest [5]. The case report outlines the importance of a thorough clinical history and consideration of the differential diagnosis for oral cavity tumours. The multidisciplinary approach to treatment ensures the most appropriate management strategy and intervention is employed.

## CASE REPORT

**Figure 1:**
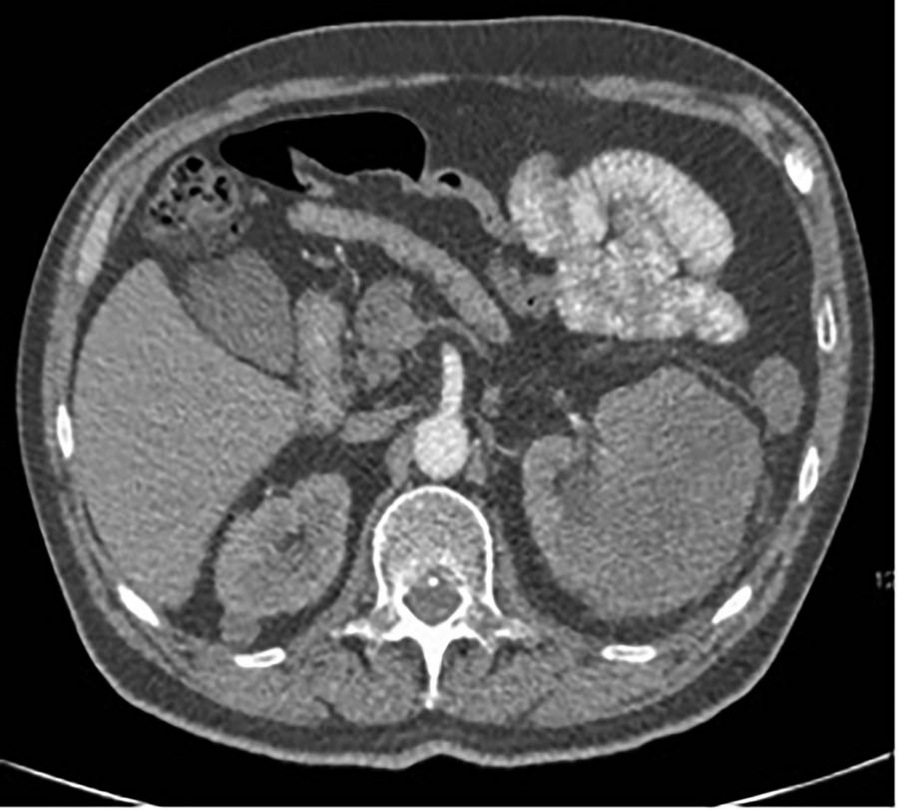
Axial CT showing a large 12-cm left renal heterogenous mass; no renal obstruction, hydronephrosis or calculus; the mass abuts the lower edge of the spleen.

**Figure 2:**
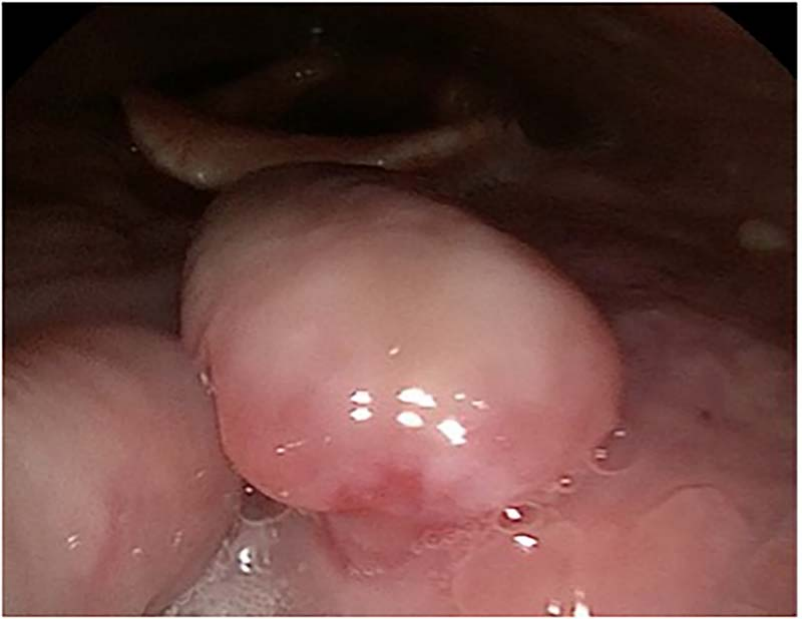
Flexible nasendoscopy image of the pedunculated RCC on the dorsum of the tongue.

**Figure 3:**
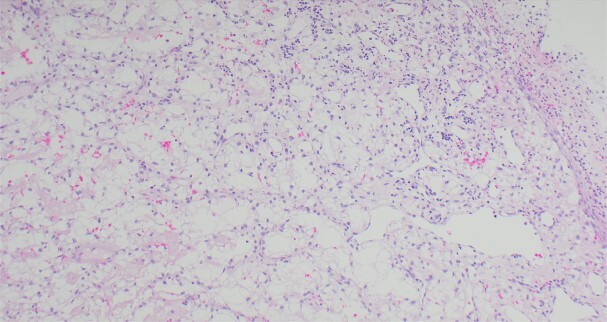
Ulcerated squamous mucosa with underlying metastatic RCC.

**Figure 4:**
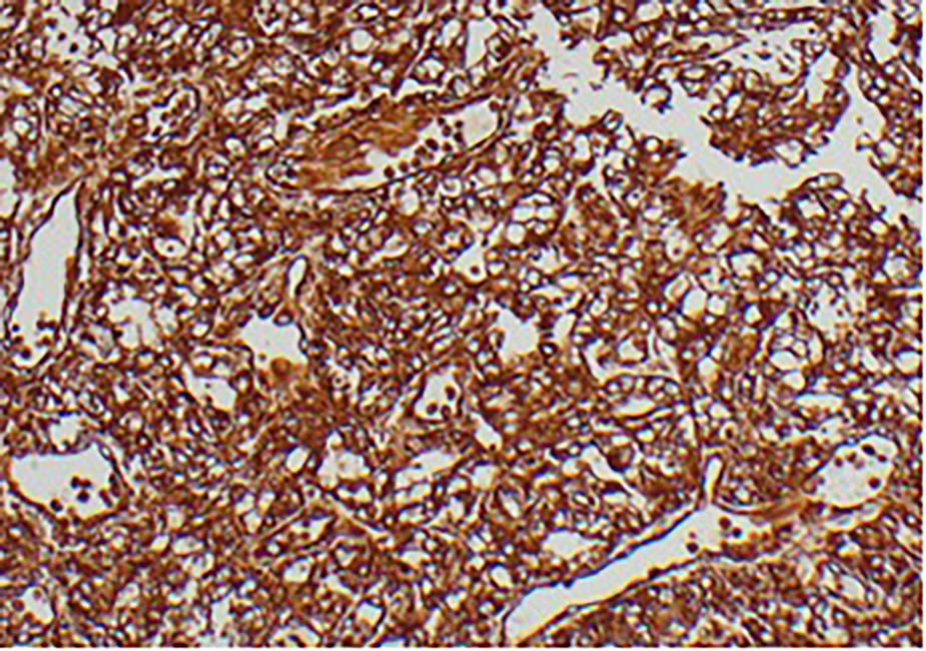
Immunohistochemical staining confirming Vimentin positivity and the presence of malignant RCC over a benign Oncoytoma.

A 63-year-old male with a medical history of hypertension and progressive dyspnoea on exertion, underwent a computed tomography (CT) angiogram for the workup of a pulmonary embolism. There was an incidental finding of a 12-cm left renal neoplasm (Fig. 1), extensive metastatic nodal abdominal disease and bilateral metastatic pulmonary disease (T3 N2 M1). He was discussed at the Urology multidisciplinary team meeting (MDT) and commenced on Sunitnib, given the dissemination of disease. Repeat imaging showed a significant reduction in size of the pulmonary metastasis. Following which, he underwent a cryo-reductive nephrectomy that confirmed a clear cell RCC 102 mm, Grade 3. Development of a tongue lesion after a year warranted Otorhinolaryngology input. Clinical assessment with flexible nasendoscopy depicted a 0.5 cm by 0.5 cm pedunculated lesion on the midline of the tongue (Fig. 2). A biopsy under local anaesthetic confirmed a clear cell RCC (Fig. 3) that was positive for AE1/AE3, Vimentin (Fig. 4) and EMA. Further CT imaging, 1 month after the biopsy confirmed left hilar nodule progression from 2.3 to 3 cm. The medical oncology MDT recommended commencing Nivolumab. Following recurrent bleeding from the tongue, radiation to the area was determined to be the best treatment modality. The patient is still clinically well and tolerating oral intake with no significant large bleeds to date. Regular medical oncology, radiation oncology, urology and Otorhinolaryngology follow-up is ongoing, however, the prognosis is poor with such disseminated disease and the primary focus is on minimising morbidity.

## DISCUSSION

The main histological subtypes of RCC are clear cell, papillary, chromophobe, collecting duct carcinoma, medullary carcinoma and unclassified [6]. Distinction between clear cell salivary tumours and RCC should be made prior to treatment, as histologically, this can be difficult to perform on standard light microscopy. A strong reaction to Vimentin will confirm this on immunohistochemical staining [7]. To find an RCC metastasis on the tongue is rare; the oral cavity consists of the anterior two thirds of the tongue, the lips, the floor of mouth, hard palate, buccal mucosa, maxillary and mandibular alveolus and the retro molar trigone. One of the theories regarding metastatic spread of RCC is that Batson’s venous plexus extends from the skull to the sacrum. This valveless system theoretically offers less resistance to the spread of tumour emboli, especially when there is an increase in intrathoracic and intra-abdominal pressure, allowing retrograde flow by-passing the pulmonary filters [8].

Primary malignant tongue lesions are considered to be SCC 90% of the time, with various subtypes identified on histopathology, including Conventional, Verrucous, Basaloid, Papillary, Sarcomatoid, Acantholytic and Adenosquamous. Notably oral SCC is associated with human papilloma virus16 in <10% of cases [9]. When we consider benign conditions in the tongue papillomas, condyloma acuminatum, granular cell tumours, keratochantoma, papillary hyperplasia and median rhomboid glossitis should be in the differential. As described previously, metastatic tongue lesions are rare and are only considered after the common diagnostic pathway for tongue lesions have been followed, with a biopsy either under local or general anaesthetic. Imaging as appropriate in conjunction with clinical and histopathology findings.

Managing these patients’ expectations and facilitating the best quality of life for them from an ENT perspective is paramount. These tumours are considered to be extremely vascular and can be associated with concurrent vascular malformations [10], therefore, making surgical intervention problematic. RCC metastases to the tongue are also known to increase in size rapidly, which can have a devastating effect on the ability to eat, drink and breathe. Surgery at this stage is usually done in the palliative setting to facilitate haemostasis or deglutination, given that distant metastasis to the tongue indicates significant disseminated disease.

Metastatic RCC can be resistant to radiotherapy and pharmacotherapy, including chemotherapy, molecularly targeted therapy or immunotherapy [11]. However, the introduction of drugs targeting the vascular endothelial growth factor pathway or the mTOR pathway has improved the prognosis of RCC [12]. As stated above, surgery can be problematic secondary to the high bleeding risk and size of the tumour, and consideration to the quality of life and prognosis should be considered prior to intervening and discussion at an MDT to tailor the most appropriate treatment.

## CONCLUSION

Treatment mainly focuses on palliation, given the degree of dissemination of RCC. Although RCC is well documented as radioresistant, radiotherapy can aid in the local symptom control [13]. Immunotherapy may improve the poor prognosis of oral metastatic RCC. Multidisciplinary care should be administered to facilitate the adequate quality of life, while minimizing morbidity.

## CONFLICT OF INTEREST STATEMENT

None declared.

## FUNDING

This research did not receive any specific grant from funding agencies in the public, commercial or not-for-profit sectors.
